# Pathologic lymph node ratio is a predictor of esophageal carcinoma patient survival: a literature-based pooled analysis

**DOI:** 10.18632/oncotarget.19258

**Published:** 2017-07-15

**Authors:** Yuming Zhao, Shengyi Zhong, Zhenhua Li, Xiaofeng Zhu, Feima Wu, Yanxing Li

**Affiliations:** ^1^ Department of Cardiothoracic Surgery, Xianning Central Hospital, The First Affiliated Hospital of Hubei University of Science and Technology, Xianning 437100, China

**Keywords:** lymph node ratio, esophageal carcinoma, survival

## Abstract

The positive lymph node ratio (LNR) has been suggested as a predictor of survival in patients with esophageal carcinoma (EC). However, existed evidences did not completely agree with each other. We sought to examine whether LNR was associated with overall survival (OS). Electronic database was searched for eligible literatures. The primary outcome was the relationship between LNR and OS, which was presented as hazard ratio (HR) with 95% confidence intervals (CIs). All statistical analyses were performed using STATA 11.0 software. A total of 18 relevant studies which involved 7,664 cases were included. Patients with an LNR of 0.3 or greater had an increased risk of death compared to those with an LNR of less than 0.3(HR = 2.33; 95% CI 2.03-2.68; P<0.01). Similarly, patients with an LNR greater than 0.5 was also associated with a decreased OS(HR = 1.95; 95% CI 1.52-2.50; P<0.01). No publication bias was found. This meta-analysis confirmed that LNR was a significant predictor of survival in patients with EC and should be considered in prognostication.

## INTRODUCTION

Esophageal cancer (EC) is one of the most common and aggressive malignancies globally, resulting in more than 400,000 deaths each year [1]. Despite the significant improvement in its diagnosis and treatment in recent decades, the prognosis of EC patients remains poor. Radical esophagectomy and subsequent lymph node (LN) dissection are considered the best option for potentially curable EC patients. The status of LN metastasis is a key factor that closely relates with the long-term survival of EC patients who underwent surgery [2-5]. Both involved LN and retrieved LN count are prognostic [6, 7]. However, insufficient LN retrieval would happen because of various factors such as physical condition of each patient, surgical or pathological diagnosing skills in clinical practice, and the number of the pathologically involved lymph nodes is significantly influenced by the number of the removed LNs.

Recent studies have proposed a superior prognostic factor, the positive lymph node ratio (LNR), for EC patients especially when insufficient LN retrieval happened [8-10]. LNR, also known as metastatic lymph node ratio (MLNR), is the ratio of the number of positive lymph nodes to the total number of dissected lymph nodes. LNR has been proposed to be used to assess the prognoses of several other solid cancers, such as colorectal cancer, non-small-cell lung cancer and pancreatic adenocarcinoma [11-13]. However, its prognostic significance in EC patients is still controversial as the existed evidence did not completely agree with each other. We sought to examine the relationship between LNR and prognosis of EC by integrating all available published data.

## RESULTS

### Eligible studies

**Figure 1:**
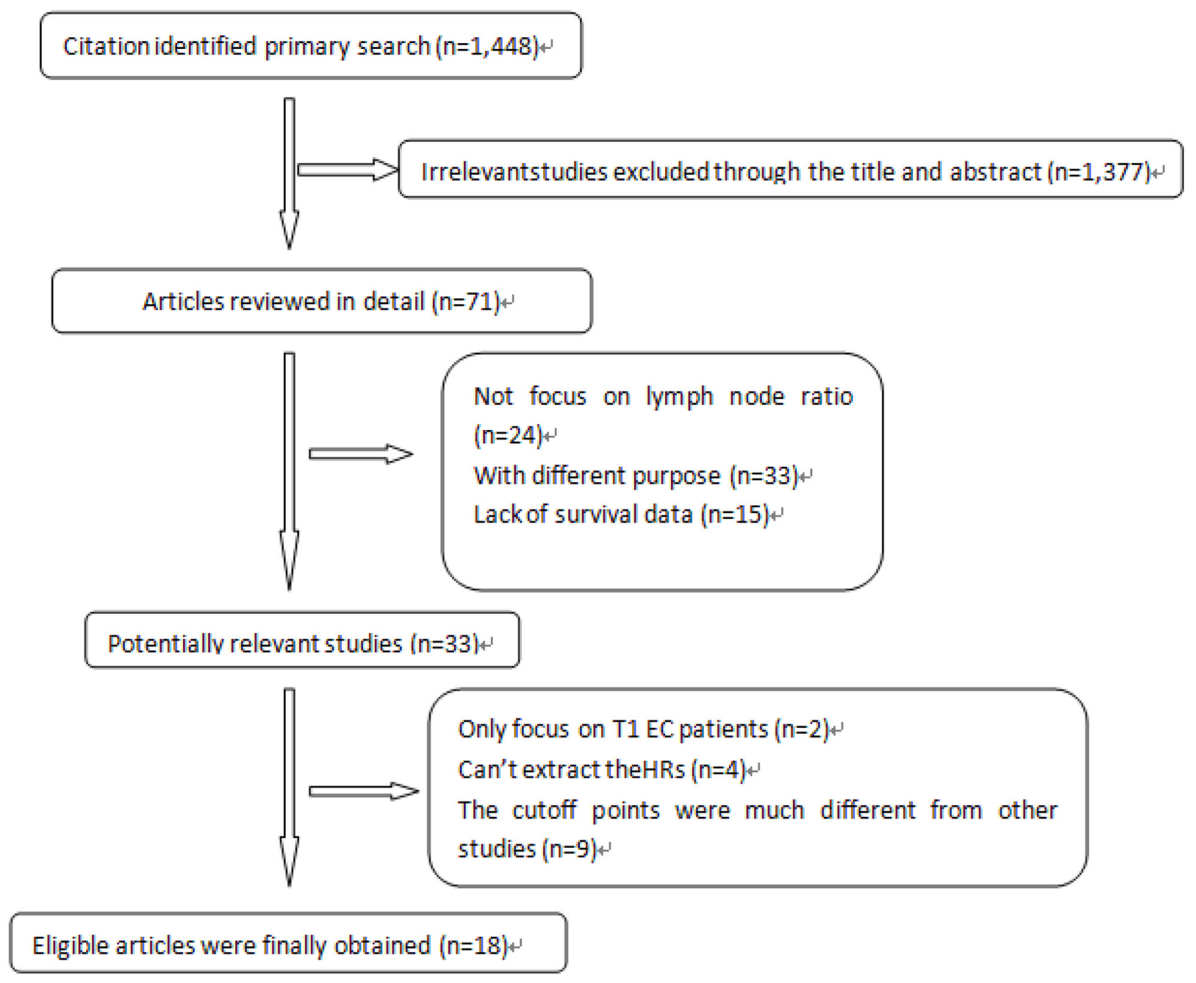
Profile summarizing the trial flow

We identified 1,448 potentially relevant records according to the search strategy. 1,376 studies were excluded after checking the corresponding title and abstract. Then the full texts of 72 articles were carefully reviewed. A total of 18 studies [8, 9, 14-29] were finally included in this meta-analysis according to the eligible criteria. Figure 1 summarized the flow chart.

### Characteristics of the included studies

Our meta-analysis was composed of 18 studies including 7,664 EC patients. These studies were conducted between 2000 and 2016. The characteristics of all included studies were summarized in Table 1. We listed the HRs and their 95% CIs under specific cut-off values of the LNRs of the collected studies in Table 2. Since the determined cut-off values of the LNRs varied from study to study, we uniformed the cutoffs for the LNRs according to the selection in the majority of studies. Therefore, 0.3(representing the range from 0.2 to 0.4) and 0.5(representing the range from 0.4 to 0.6) were selected as the cutoff values of LNR in our study.

**Table 1:** Characteristic of the included studies

First author	Year	Patient age	T-stage	N-stage	Case number(n)	Country	Cut-off point	Resected nodes (median/average)	Surgical approach
Castigliano	2012	-	Ttis-4	N0-3	347	USA	0.1,0.2,0.3	14	Thoracotomy/Nonthoracotomy
Chen	2015	56	T1-4	N0-3	496	China	0.15,0.3	7	_
Zhang	2014	62	T1-4	N0-3	337	China	0.3,0.6	_	_
Lagergren	2015	64	T0-4	_	606	England	0.14,0.37	_	transhiatal or transthoracic esophagectomy
Wang	2015	60.6	_	N0-3	209	China	0.2	_	_
Wu	2013	_	T0-4a	_	205	China	0.1,0.2,0.3,0.4,0.5	10.2	two-field lymph node dissection/three-field lymph node dissection
Tang	2013	_	T1-4	_	170	China	0.32	_	_
Sandick	2001	_	T1-4	N0-3	111	Netherlands.	0.3	12	transhiatal technique
Wijnhoven	2007	63	T1-4	_	292	Australia	0.2	11	transhiatal technique
Zhang	2016	_	T1-4	N0-3	389	China	0.3,0.6	17.5	_
Liu	2010	54.8	_	_	1325	China	0.25,0.5	21.2	transhiatal or transthoracic esophagectomy
Shao	2016	_	T0-4a	N0-3	916	China	0.1,0.35	12	transthoracic esophagectomy
Zafirellis	2002	_	T0-4	N1-2	156	China	0.2	13	thoracoabdominal incision
Tan	2014	57	T0-4	N0-3	700	China	0.25	16.4	tri-incisional approach
Bogoevski	2008	61	T1-4	N0-1	235	Germany	0.11,0.33	18	transhiatal or transthoracic esophagectomy
Hsu	2009	63.8	T1-4	N0-1	488	Taiwan	0.2	22	Tri-incisional/Transhiatal/Thoracoabdominal/IVOR Lewis
Mariette	2008	58	T1-3	N0-1	509	Australia	0.2	_	transthoracic en bloc esophagectomy
Wilson	2008	62	T1-3	N0-3	173	USA	0.25,0.5	_	Tri-incisional/Transhiatal/Thoracoabdominal/IVOR Lewis

**Table 2:** Summary table of HRs (95% CI) and HR calculation

First author	Year	HR	LL	UL	Cut-point
Castigliano	2012	2.04	0.06	67.5	0.3
Chen	2015	2.35	1.64	3.78	0.3
Zhang	2014	2.25	1.03	4.91	0.3
		2.564	1.33	4.942	0.6
Lagergren	2015	2.22	1.31	3.76	0.37
Wang	2015	3.059	2.114	4.426	0.2
Wu	2013	2.72	1	7.38	0.3
		2.315	0.775	6.912	0.5
Tang	2013	2.44	1.79	3.33	0.32
Sandick	2001	1.87	0.72	4.81	0.3
Wijnhoven	2007	1.98	1.029	3.79	0.2
Zhang	2016	2.36	1.0135	5.5	0.3
		2.82	1.578	5.04	0.6
Liu	2010	1.584	1.05	2.38	0.25
		1.644	1.143	2.363	0.5
Shao	2016	2.08	1.31	3.3	0.35
Zafirellis	2002	4.55	2.94	7.14	0.2
Tan	2014	1.94	1.45	2.59	0.25
Bogoevski	2008	1.656	0.98	2.81	0.33
Hsu	2009	2.97	2.096	4.196	0.2
Mariette	2008	2.65	2.02	3.48	0.2
Wilson	2008	1.11	0.54	2.27	0.25
		1.53	0.8	2.94	0.5

### Quantitative data synthesis

**Figure 2:**
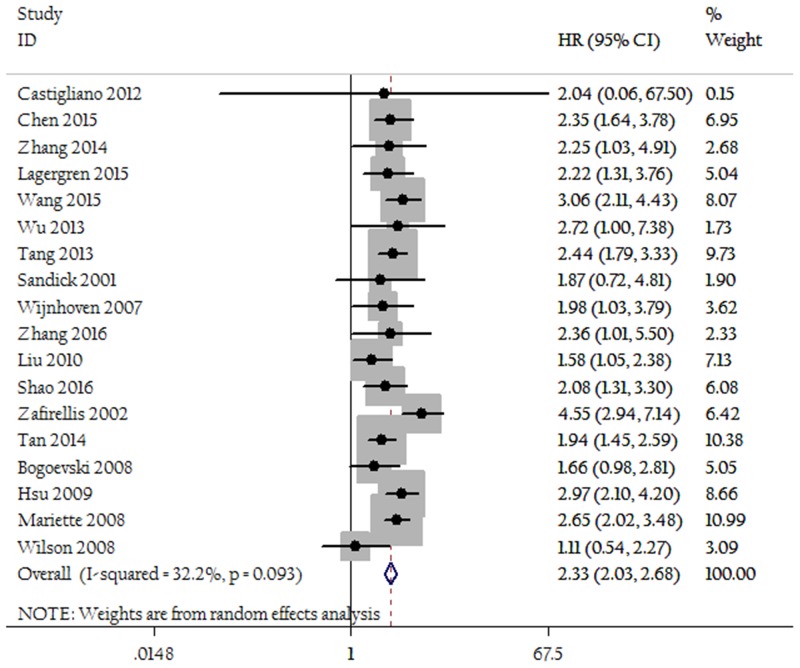
Forest plots show the association between LNR of 0.3 and overall survival

**Figure 3:**
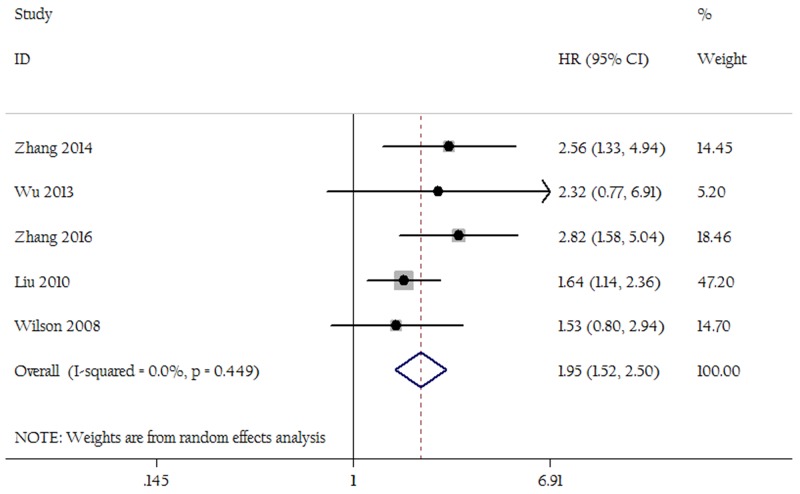
Forest plots show the association between LNR of 0.5 and overall survival

As shown in Figure 2, patients with an LNR of 0.3 or greater had an increased risk of long-term deaths compared to those with a LNR less than 0.3(HR=2.33; 95% CI 2.03-2.68; P<0.01). There was no significant heterogeneity (I^2^=32.2%, P=0.09). As shown in Figure 3, patients with an LNR of 0.5 or greater was also associated with decreased OS (HR=1.95; 95% CI 1.52-2.50; P<0.01; heterogeneity test, I^2^=0.0%, P=0.45). Sensitivity analyses did not change the trends. After the exclusion of results from studies using multivariate analysis, patients with an LNR of 0.3 or greater also had an increased risk of deaths compared to those with an LNR less than 0.3 (HR=2.27; 95% CI 1.93-2.26; P<0.05). All studies using an LNR of 0.5 as cut-off were multivariate setting, thus sensitivity analysis was unavailable.

### Publication bias

**Figure 4:**
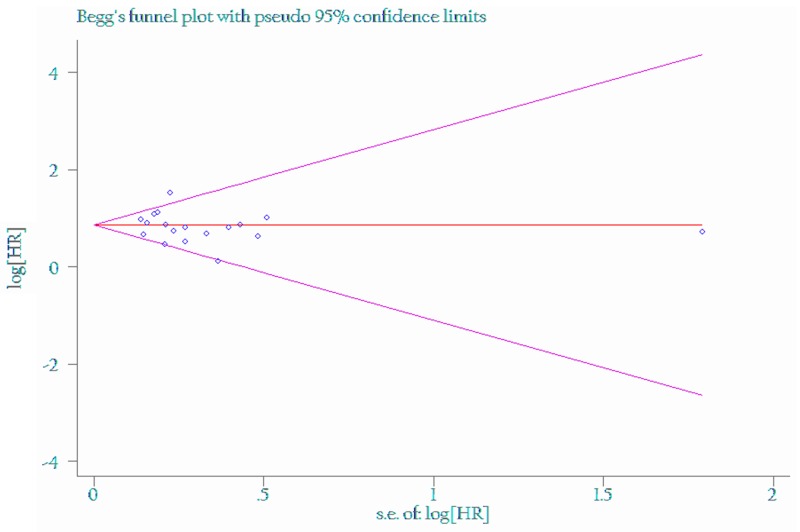
Funnel plot of the association between LNR of 0.3 and overall survival

**Figure 5:**
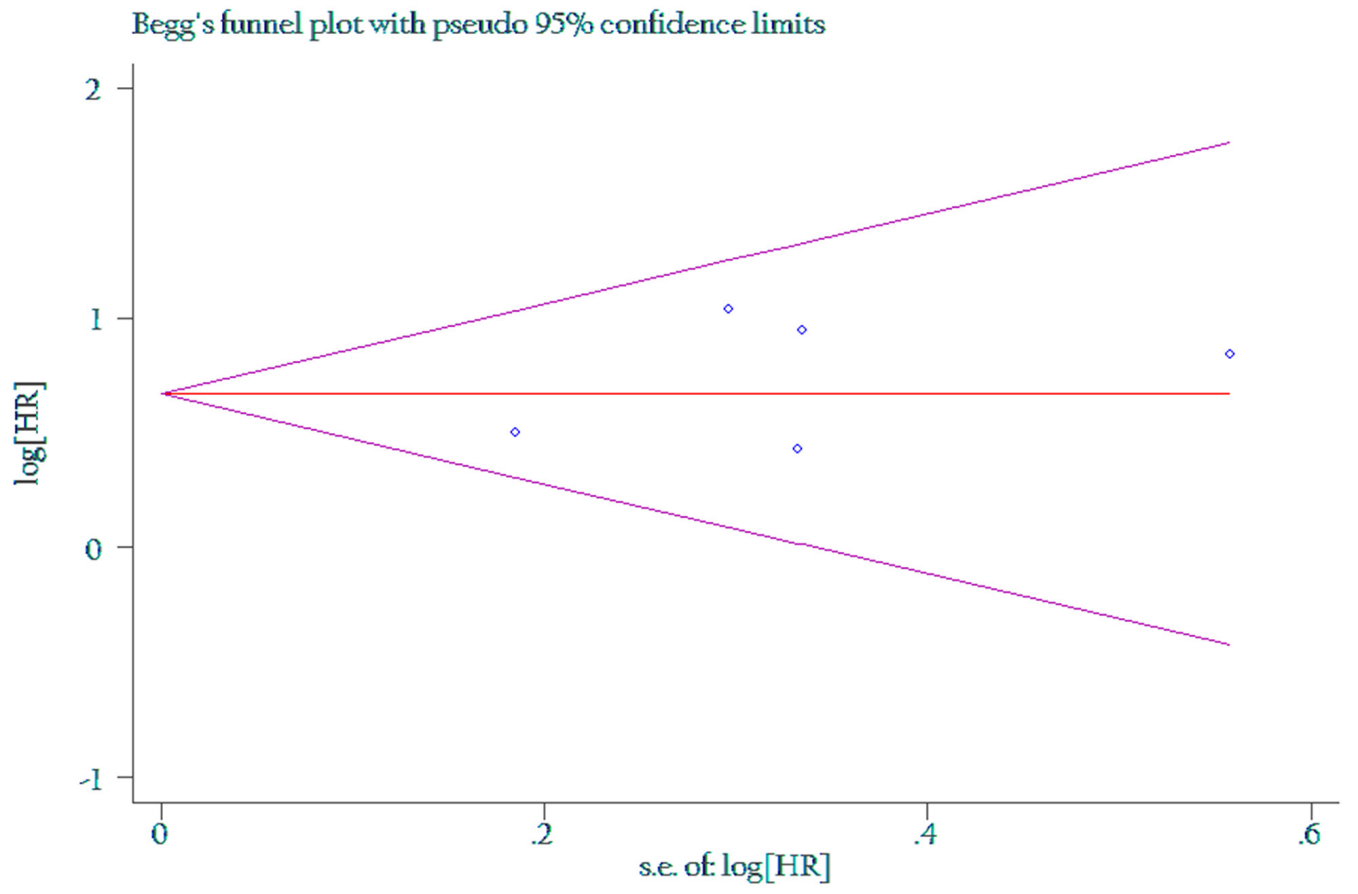
Funnel plot of the association between LNR of 0.5 and overall survival

The funnel plot and Egger’s test were performed for the overall comparison. No obvious visual asymmetry was observed in funnel plots (Figures 4 and 5) for OS, and the P values of the Egger’s test were all greater than 0.05.

## DISCUSSION

Tumor staging system has multiple roles: prognosis prediction, determination of treatment strategy, and the adjustment for the comparison of treatment effects. The status of LN metastasis in patients with esophageal cancer has been considered a pivotal prognostic factor [30, 31]. Since the accuracy of the number of metastatic LNs depends on the number of nodes removed, the 7th edition AJCC TNM staging system suggested more than 12 nodes should be sampled. To allow best predictive outcome, Peyre et al. have suggested that the number of removed nodes should range from 23 to 29 [32]. Considering the total number of LNs retrieved may be affected by many factors, LNR emerged as a more simple strategy to make up for insufficient LN retrieval. Furthermore, emerging evidence indicates that LNR showed better prognostic value than metastatic LN number for esophageal cancer [17, 33, 34].

Combining the available data of 18 studies, our results confirmed that no matter the cutoff point was 0.3 or 0.5, higher LNR is significantly associated with a poorer survival of esophageal cancer. Observations on other malignancies from many other studies were consistent with ours that LNR could be a prognostic factor. Sun and his coworkers [35] published a meta-analysis involving12 observational studies, showing that higher LNR was significantly associated with a poorer survival of NSCLC(OS HR=1.93; 95% CI 1.64-2.28, DDS HR=1.82; 95% CI 1.55-2.14). A previous meta-analysis also indicated that LNR was a prognostic factor with regard to overall survival for breast cancer and colorectal cancer [36, 37].

Since the different extent of lymph node dissection and the pathological type of esophageal cancer, various cutoffs of LNR have been used in different studies. For breast cancer, Liu et al. suggested that the suitable cutoff point were 0.2 and 0.65 [36]. In the present study, we determined 0.3 and 0.5 as cutoff values since they were used in most of the articles.

This is the first study to comprehensively answer the prognostic role of LNR in EC patients. However, there are several limitations. First, it was based on retrospective analyses; prospective analysis is needed to further clarify these issues. Second, the different cutoff values for defining high LNR may have contributed to heterogeneity. Third, we can’t rule out the effects of the chemotherapeutic agents or radiotherapy after surgery or concurrent radiochemotherapy (CRT) before surgery as such information was not provided in the original reports. In addition, we cannot study adenocarcinoma and squamous cell carcinoma, or the location of the tumor separately for insufficient information provided by primary studies; we can distinguish the prognostic roles of LNR on these subtypes. Further studies are warranted.

In conclusion, this meta-analysis confirmed that LNR was a strong predictor of survival in patients with EC. The appropriate incorporation of LNR into the prognostic system or the treatment determination (such as postoperative radiotherapy) of EC should be discussed.

## MATERIALS AND METHODS

### Literature search

All relevant articles were retrieved by searching PubMed, Embase and the Central Registry of Controlled Trials of the Cochrane Library using a combination of the terms: (“EC or “esophageal carcinoma” or “esophageal cancer”) and ratio. No restriction by language or year was set in the search. The last research time was October 23, 2016. References from relevant articles, including review papers, were also reviewed.

### Inclusion criteria and exclusion criteria

Eligible studies should meet the following criteria: (1) studies which evaluated the association between LNR and prognosis of EC. (2) studies published in English or Chinese regardless of publication time.(3) the original papers containing enough data. Studies failed to meet the inclusion criteria will be excluded.

### Data collection

Two authors searched eligible studies and extracted information independently and finally negotiate to reach consensus. Details of publication characteristics such as first author’s name, publication year, middle/mean age of study sample, sample size, T stage, N stage, cutoff point and hazard ratio (HR) and the corresponding 95% CI were collected from each eligible publication. If the results of univariate and multivariate analysis were both reported in a study, the former was chosen. If precise HR (95% CI) were provided in the study, we used them directly, otherwise we used Engauge Digitizer version 2.11 software to extract relevant numerical value from survival curves and calculate the HR(95% CI) when only Kaplan-Meier survival curves were provided [38, 39].

### Statistical analysis

Cochran’s Q-statistic test and I^2^ test were used to calculate the heterogeneity. As to Q-statistic, P < 0.05 was considered to have statistical significance. For I^2^ statistics, I^2^ < 25% indicated no heterogeneity; I^2^ = 25–50% indicated moderate heterogeneity; and I^2^ > 50% indicated strong heterogeneity [40, 41]. A random effects model was applied to minimize the impact of any potential bias. Subgroup analysis and sensitivity analysis were performed. Sensitivity analyses were conducted to assess the strength of our findings by excluding one study at a time. Publication bias was investigated by funnel plots and by Egger’s test. All statistical analyses were performed with STATA 11.0 software.

Of note, patients with LNR=0 was the reference group in many studies. We used LNR=0 as the link to calculate relevant HR and the standard error for the log HR was $\text{SE}\left(\text{log}{\text{HR}}_{\text{AB}}\right)=\sqrt{\text{SE}{\left(\text{log}{\text{HR}}_{\text{AC}}\right)}^{2}+\text{SE}{\left(\text{log}{\text{HR}}_{\text{BC}}\right)}^{2},}$ in which log HRAC was the log HR for the direct comparison of patients who with LNR equal to or greater than the cut-points versus those who with LNR=0, and log HRBC were log HR for the direct comparison of patients who with LNR between 0 and a number less than the cut-points versus those who with LNR=0. SE (logHR) was the standard error of the log HR for the direct comparisons.
